# Juvenile idiopathic arthritis polygenic risk scores are associated with cardiovascular phenotypes in early adulthood: a phenome-wide association study

**DOI:** 10.1186/s12969-022-00760-0

**Published:** 2022-11-19

**Authors:** Sarah L. N. Clarke, Hannah J. Jones, Gemma C. Sharp, Kayleigh E. Easey, Alun D. Hughes, Athimalaipet V. Ramanan, Caroline L. Relton

**Affiliations:** 1 MRC Integrative Epidemiology Unit, University of Bristol, Oakfield House, Oakfield Grove, Bristol, UK; 2 School of Population Health Sciences, Bristol Medical School, University of Bristol, Bristol, UK; 3 Department of Paediatric Rheumatology, Bristol Royal Hospital for Children, Bristol, UK; 4 National Institute for Health Research Bristol Biomedical Research Centre at University Hospitals Bristol and Weston NHS Foundation Trust and the University of Bristol, Bristol, UK; 5 School of Psychology, University of Exeter, Exeter, UK; 6 School of Social Sciences, University of the West of England, Bristol, UK; 7 Department of Population Science and Experimental Medicine, UCL Institute of Cardiovascular Science, University College London, London, UK; 8 MRC Unit for Lifelong Health and Ageing at UCL, University College London, London, UK; 9 School of Translational Health Sciences, Bristol Medical School, University of Bristol, Bristol, UK

**Keywords:** Genetics, Juvenile idiopathic arthritis, Cardiovascular, ALSPAC

## Abstract

**Background:**

There is growing concern about the long-term cardiovascular health of patients with juvenile idiopathic arthritis (JIA). In this study we assessed the association between JIA polygenic risk and cardiovascular phenotypes (cardiovascular risk factors, early atherosclerosis/arteriosclerosis markers, and cardiac structure and function measures) early in life.

**Methods:**

JIA polygenic risk scores (PRSs) were constructed for 2,815 participants from the Avon Longitudinal Study of Parents and Children, using the single nucleotide polymorphism (SNP) weights from the most recent JIA genome wide association study. The association between JIA PRSs and cardiovascular phenotypes at age 24 years was assessed using linear and logistic regression. For outcomes with strong evidence of association, further analysis was undertaken to examine how early in life (from age seven onwards) these associations manifest.

**Results:**

The JIA PRS was associated with diastolic blood pressure (β 0.062, 95% CI 0.026 to 0.099, *P* = 0.001), insulin (β 0.050, 95% CI 0.011 to 0.090, *P* = 0.013), insulin resistance index (HOMA2_IR, β 0.054, 95% CI 0.014 to 0.095, *P* = 0.009), log hsCRP (β 0.053, 95% CI 0.011 to 0.095, *P* = 0.014), waist circumference (β 0.041, 95% CI 0.007 to 0.075, *P* = 0.017), fat mass index (β 0.049, 95% CI 0.016 to 0.083, *P* = 0.004) and body mass index (β 0.046, 95% CI 0.011 to 0.081, *P* = 0.010). For anthropometric measures and diastolic blood pressure, there was suggestive evidence of association with JIA PRS from age seven years. The findings were consistent across multiple sensitivity analyses.

**Conclusions:**

Genetic liability to JIA is associated with multiple cardiovascular risk factors, supporting the hypothesis of increased cardiovascular risk in JIA. Our findings suggest that cardiovascular risk is a core feature of JIA, rather than secondary to the disease activity/treatment, and that cardiovascular risk counselling should form part of patient care.

**Supplementary Information:**

The online version contains supplementary material available at 10.1186/s12969-022-00760-0.

## Background

Juvenile idiopathic arthritis (JIA) is the most common rheumatic disorder of childhood with an estimated prevalence of 32.6/100,000 in Europeans [1]. It is characterised by chronic (> 6 weeks) idiopathic onset of joint inflammation and is associated with considerable morbidity. Although some patients enter remission over time, more than half do not achieved remission after 10 years of active disease [2].

Risk factors for cardiovascular disease (CVD) such as obesity, sedentary lifestyle, hypertension, smoking, dyslipidaemia and diabetes are well characterised [3]. However there is substantial evidence of a role of systemic inflammation in atherosclerosis [4]. The increased risk of CVD in patients with systemic autoimmune disorders such as rheumatoid arthritis (RA) and systemic lupus erythematosus (SLE) is well established [5] and is now captured within cardiovascular risk scoring calculators, such as QRISK3 [6]. European guidance also exists for risk factor assessment and modification of cardiovascular risk in adult inflammatory joint diseases [7]. Accordingly, there is concern about the cardiovascular health of JIA patients given their early age of onset of systemic inflammation [8]. However, there is currently no guidance on cardiovascular risk factor assessment of children or adults with JIA. Evidence from cross-sectional and case–control studies suggests JIA is also associated with adverse cardiovascular risk factors or markers of early atherosclerosis [9–13]. However, such studies are at risk of confounding by disease duration, disease activity and treatment.

The wider availability of genomic data has enabled observational associations to be further examined using methods less susceptible to bias due to confounding. Polygenic risk scoring quantifies an individual’s genetic liability to a disease or phenotype, with potential use in disease risk prediction and stratification [14]. We recently highlighted the genetic overlap between rheumatoid factor negative polyarticular and oligoarticular JIA, and coronary artery disease in adulthood [15]. In the current study we extend this work to assess whether genetic liability to JIA (all subtypes), as captured by polygenic risk scores (PRSs), is associated with specific cardiovascular phenotypes in early adulthood—cardiovascular risk factors, markers of early atherosclerosis, and measures of cardiac structure and function. We then examined the cardiovascular phenotypes strongly associated with JIA PRSs in a longitudinal analysis, to identify how early in childhood (from age seven onwards) these associations manifest.

## Methods

### Study population

The Avon Longitudinal Study of Parents and Children (ALSPAC) is a prospective, longitudinal birth cohort [16–18]. Pregnant women with an expected dates of delivery between 1^st^ April 1991 and 31^st^December 1992 and residing in Avon, Southwest England, were invited to enrol in the study. The initial recruitment plus subsequent catch-up campaigns, resulted in a total sample of 15,454 pregnancies, from which 14,901 children were born who survived to one year of age. Children within the ALSPAC cohort have detailed health data throughout childhood and early adulthood in the form of questionnaires, clinic measures and biological samples [16–18]. The ALSPAC study website contains details of all the data that is available through a fully searchable data dictionary and variable search tool (http://www.bristol.ac.uk/alspac/researchers/our-data/) [19]. Study data were collected and managed using REDCap electronic data capture tools hosted at the University of Bristol [20]. REDCap (Research Electronic Data Capture) is a secure, web-based software platform designed to support data capture for research studies.

### Polygenic risk score for JIA

Children from the ALSPAC cohort were genotyped using the Illumina HumanHap550 quad chip genotyping platforms as previously described [21]. Genetic data is currently available for 7,977 children from the ALSPAC cohort. PRSs were constructed using the SNP weights from the most recent JIA genome wide association study (GWAS) [22]. This GWAS dataset includes 3,305 JIA cases of all disease subtypes genotyped using Illumina Infinium CoreExome and Infinium OmniExpress arrays, and 9,196 healthy controls genotyped using the Illumina Infinium CoreExome array. Further information on the genetic datasets can be found in the Supplementary Methods. Weighted PRSs were generated using the PRSice2 polygenic risk score software [23] at a range of *P* value thresholds (*P* ≤ 0.01 to *P* ≤ 5 × 10^–8^, Supplementary Table 1). SNPs within a 250 kb window and in linkage disequilibrium (LD) at an r^2^ threshold > 0.1 were clumped. For the primary analysis we used the PRSs generated using a *P* value threshold of 1 × 10^–5^ as this is the threshold typically employed to identify regions of suggestive association with the trait of interest in GWASs (Supplementary Table 2). The additional PRSs were used in sensitivity analyses. Given the complex LD within the major histocompatibility complex (MHC) region and its substantial influence in many inflammatory and autoimmune disorders, we generated two different sets of PRSs. For the main analysis, we removed the extended MHC region (chromosome 6: 25-34 Mb) from the datasets and represented it using one SNP from this region which had the lowest *P* value in the JIA dataset and was also present within the ALSPAC dataset (rs115649989). As a sensitivity analysis we omitted the extended MHC region (chromosome 6: 25-34 Mb) entirely. All PRSs were standardised using z-score transformation to allow comparison.

### Validation of the JIA PRS

Given the lack of International Classification of Diseases coding for JIA within the ALSPAC dataset, we validated the JIA PRS using a positive and negative control design. We used one variable expected to show positive association with JIA polygenic risk (positive control) and three variables expected to have no association with JIA polygenic risk (negative controls). For the positive control, we exploited the high degree of genetic overlap between autoimmune disorders to assess the association between JIA PRS and diagnosis of any autoimmune disorder by age 24 years. This variable was derived from those patients who reported a diagnosis (by self or doctor), ever versus never, of any of psoriasis, Crohn’s disease, ulcerative colitis, ankylosing spondylitis, psoriatic arthritis, spondyloarthropathy, rheumatoid arthritis, Sjögren’s syndrome, systemic lupus erythematosus, Grave’s disease, multiple sclerosis, Hashimoto’s thyroiditis or type 1 diabetes at age 24 years. As negative controls we examined the association between JIA PRS and pigeon infestation in the home (ever/never), mouse infestation in the home (ever/never) and wasp/bee sting by age three years (ever/never). We examined the association between JIA PRS, and control variables using both univariate and multivariable logistic regression (adjusted for the first ten ancestry-informed principal components, sex and age).

### Outcome variables

Full details of the methods used to obtain the outcome variables used in this analysis are available in the Supplementary Methods.

#### Cardiovascular risk factors

We used a range of traditional cardiovascular risk factor measures which were assessed at seven clinic visits between the ages of seven and 24 years. These included resting systolic and diastolic blood pressure (BP), body mass index (BMI), fat mass indexed to height (FMI), and waist circumference. Blood measures of systemic inflammation (high sensitivity C-reactive protein [hsCRP] and glycoprotein acetylation), lipid profiles (total cholesterol, low density lipoprotein cholesterol [LDL], high density lipoprotein cholesterol [HDL], apolipoprotein A-I [Apo-AI], apolipoprotein B [Apo-B], Apo-B:AI ratio and triglycerides), metabolic measures (insulin, glucose and Homeostasis Model Assessment 2 insulin resistance index [HOMA2_IR, Diabetes Trials Unit, Oxford]) were also assessed. For blood samples taken at age 15 years onwards participants fasted overnight or for at least six hours prior to their clinic visit; blood samples at earlier time points were non-fasting.

#### Early atherosclerosis/arteriosclerosis markers

Carotid intima media thickness (cIMT) measures the thickness of the tunica intima and tunica media layers of the carotid artery and is considered an indicator of subclinical atherosclerosis [24]. Pulse wave velocity (PWV) measures the speed of propagation of the blood pressure pulse along a segment of an artery, calculated based on time to travel between two locations and the distance between them. PWV is influenced by the elastic properties of a blood vessel, and therefore is an assessment of arterial stiffness [25]. cIMT and PWV both correlate with future CVD risk and were measured as part of routine research clinic visits.

#### Cardiac structure and function

Cardiac structure and function measures were derived from echocardiographic measures obtained during research clinic visits. Cardiac systolic function was assessed using ejection fraction (EF) and fractional shortening (FS). Cardiac diastolic function was examined using mitral E/A ratio, E/e’ ratio and left atrial diameter. High left ventricular mass (LVM) has been shown to be an independent predictor of increased CV morbidity and mortality in adult general [26] and adult hypertensive populations [27]. LVM was measured according to American Society of Echocardiography Guidelines [28] and indexed to height^2.7^.

### Statistical analysis

Multivariable linear regression and logistic regression were used to examine the association between JIA PRS, and continuous and dichotomous cardiovascular phenotypes, respectively. Density plots were visually inspected and variables which were strongly skewed underwent natural log transformation to achieve approximately normal distributions prior to analysis. Extreme outlier values (greater than three times the interquartile range) were removed prior to analysis and all continuous outcome variables were standardised using z-score transformation to allow comparison. All analyses were adjusted for sex and the first ten ancestry-informed principal components. All analysis was undertaken using R (version 4.0.2) [29] in RStudio (version 1.3.1073) [30]. Effect estimates for continuous outcomes are presented as β coefficients and represent standard deviation (SD) change in the outcome per SD increase in JIA PRS. β coefficients for dichotomous outcome variables were exponentiated to represent odds ratio (OR) per SD change in JIA PRS.

**Fig. 1:**
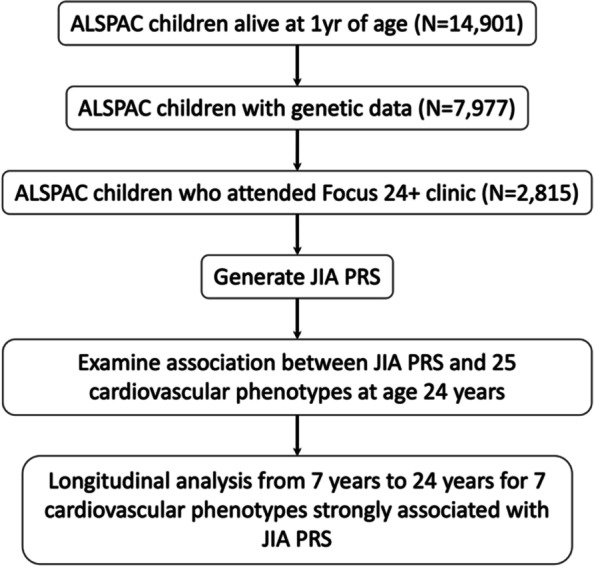
Flow diagram of study design. ALSPAC, Avon Longitudinal Study of Parents and Children; JIA, juvenile idiopathic arthritis; PRS, polygenic risk score

The primary analysis used outcome measures from the “Focus24^+^” clinic—the research clinic visit at age 24 years. For any outcome variables strongly associated with JIA PRS, we then undertook a retrospective analysis examining the association between JIA PRS and the given outcome variable at earlier clinic visits at age 17 years, 15 years, 13 years, 11 years, 9 years and 7 years. See Fig. 1 for a flow design of study design.

## Results

### Cohort description

Of the 7,977 offspring from the ALSPAC cohort with a PRS measure, 2,815 attended the “Focus 24^+^” clinic and were included in further analysis (mean age 24.5 years, range 22.5–26.4 years). The sample size for each cardiovascular phenotype ranged from 1,432 to 2,799. The descriptive statistics for the cohort are in Table 1 and description of missing data can be found in Supplementary Table 3.

**Table 1:** Descriptive characteristics of the participants with genotyping data who attended the “Focus24 + ” clinic

Category	Outcome	Sample size	Mean (SD)^a^
Demography	Age, months	2815	293.96 (9.46)
	Sex, female	2815	1720 (61.1)^a^
Control variables	Autoimmune disease, ever versus never	1964	117 (6.0)^a^
	Pigeon infestation, ever versus never	2540	282 (11.1)^a^
	Mice infestation, ever versus never	2540	396 (15.6)^a^
	Bee/wasp sting, ever versus never	2435	757 (31.1)^a^
Blood pressure	Systolic BP, mmHg	2799	116.03 (11.40)
	Diastolic BP, mmHg	2798	66.73 (7.85)
	Hypertension, > 140/90 mmHg	2798	7 (0.3)^a^
	Isolated diastolic hyerptension, < 140/ > 90 mmHg	2791	7 (0.3)^a^
	High normal blood pressure, > 120/80 mmHg	2798	138 (4.9)^a^
Anthropometry	Waist circumference, cm	2776	81.34 (12.39)
	BMI, kg/m^2^	2782	24.83 (4.85)
	BMI category	2782	
	Underweight, < 18.5 kg/m^2^		78 (2.8)^a^
	Normal, 18.5–24.9 kg/m^2^		1643 (59.1)^a^
	Overweight or obese, 25–39.9 kg/m^2^		1061 (38.1)^a^
	Obese, 25–29.9 kg/m^2^		353 (12.7)^a^
	FMI, kg/m^2^	2697	7.97 (3.73)
Blood biomarkers	hsCRP, mg/L	2056	1.36 (1.50)^b^
	Glycoprotein acetylation, mmol/L	2338	1.23 (0.17)
	Triglycerides, mmol/L	2309	0.94 (0.41)
	HDL, mmol/L	2339	1.56 (0.42)
	LDL, mmol/L	2337	2.44 (0.75)
	Total cholesterol, mmol/L	2338	4.44 (0.83)
	Apo-AI, g/L	2336	1.46 (0.21)
	Apo-B, g/L	2336	0.68 (0.15)
	Apo-B:AI	2336	0.47 (0.11)
	Glucose, mmol/L	2321	3.90 (0.32)
	Insulin, mU/L	2264	8.33 (4.53)
	HOMA2_IR	2245	0.87 (0.47)
Early atherosclerosis/arteriosclerosis	cIMT, mm	1566	0.46 (0.05)
	Pulse wave velocity, m/sec	1699	6.22 (0.94)
Cardiac structure and function	Ejection fraction, %	1433	63.75 (6.82)
	Fractional shortening, %	1432	34.85 (5.08)
	Mitral E/A	1597	1.97 (0.53)
	E/e'	1519	5.99 (1.07)
	Left atrial diameter, cm	1519	3.13 (0.42)
	LVMI, g/m^2.7^	1461	30.53 (6.52)

*Apo-AI* Apolioprotein-AI, *Apo-B* Apolipoprotein-B, *BMI* Body mass index, *BP* Blood pressure, *cIMT* carotid intima-media thickness, *hsCRP* high sensitivity C-reactive protein, *FMI* Fat mass index, *HOMA2_IR* Homeostasis Model Assessment 2 insulin reistance index, *HDL* High density lipoprotein cholesterol, *LDL* Low density lipoprotein cholesterol, *LVMI* Left ventricular mass index

^a^ For measures which are not continuous N (%) is reported.

^b^ For log hsCRP the geometric mean (SD) is 0.805 (2.81).

### Validation of JIA PRS in the ALSPAC cohort

**Fig. 2:**
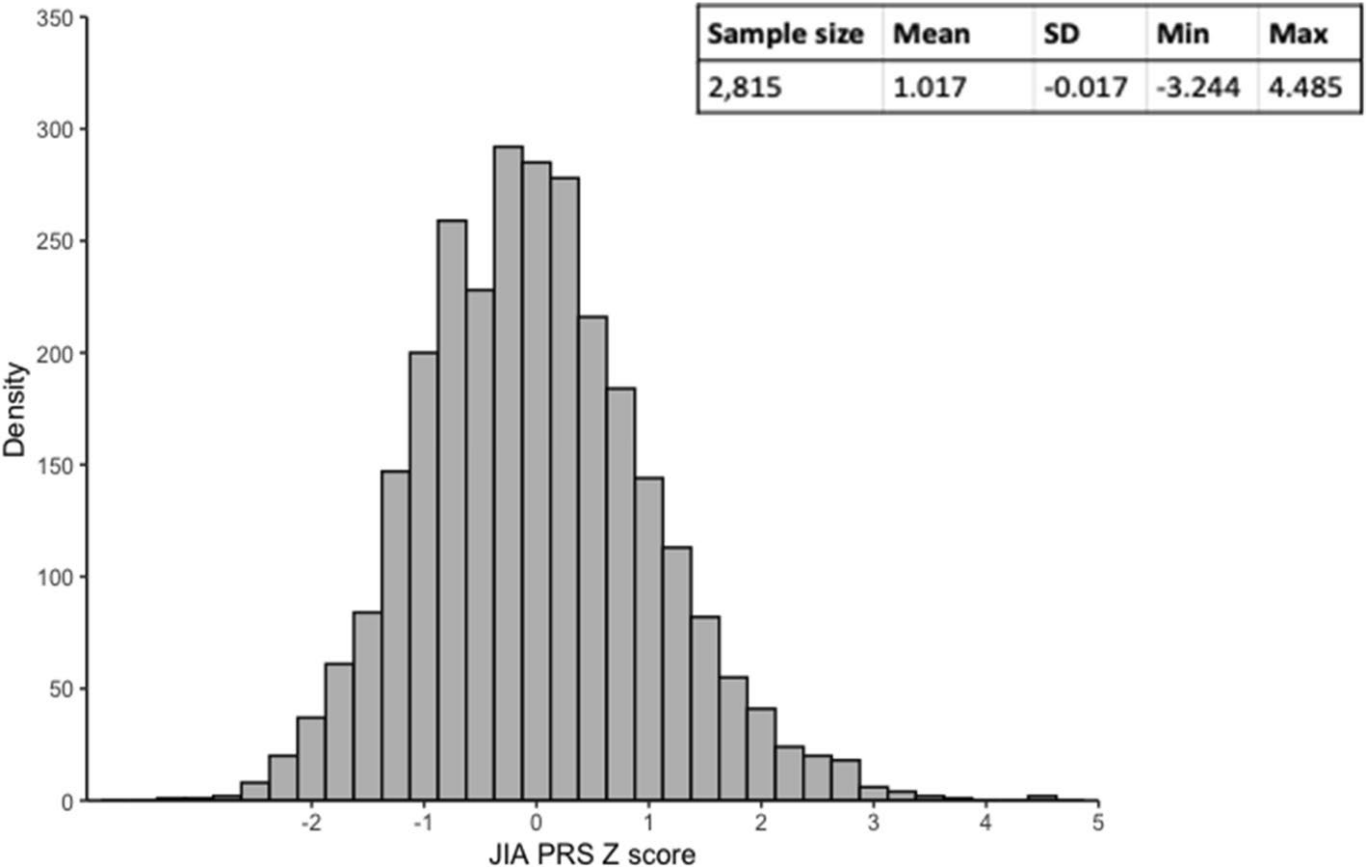
Distribution of JIA PRS at age 24 years. SD, standard deviation; PRS, polygenic risk score

**Fig. 3:**
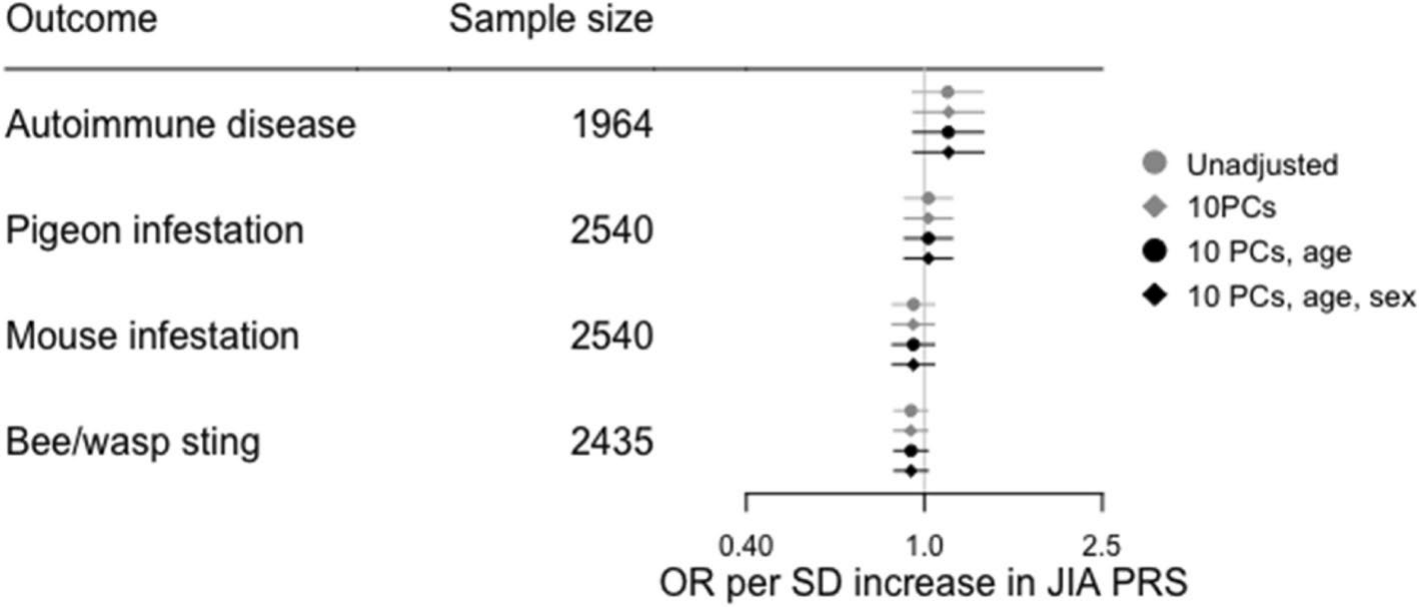
Association between JIA PRS and control variables – diagnosis of autoimmune disease (ever versus never, positive control), home infested by pigeons (ever versus never, negative control), home infested by mice (ever versus never, negative control), bee/wasp sting by age 3 years (ever versus never, negative control). JIA, juvenile idiopathic arthritis; OR, odds ratio; PCs, principal components; PRS, polygenic risk score; SD, standard deviation

The distribution of JIA PRS derived using 57 SNPs with a *P* value threshold of 1 × 10^–5^ (Supplementary Table 2) in the ALSPAC cohort is shown in Fig. 2. To examine how well the JIA PRS may capture genetic liability to JIA, we examined the association between the JIA PRS and diagnosis of an autoimmune disorder (ever versus never) based on questionnaire data at age 24 years. Of the 1,964 participants for whom data on autoimmunity was available, 117 (6%) reported a diagnosis of at least one autoimmune disorder (Table 1). There was some evidence that increasing JIA PRS was associated with increased risk of previous diagnosis of an autoimmune disorder (Supplementary Table 4). We also examined the association between each PRS and three negative control variables, with sample size 2,435 to 2,540 (Table 1) and found no strong evidence of an association between JIA PRS and any negative control variable (Fig. 3, Supplementary Table 4).

### Associations between JIA and cardiovascular phenotypes at age 24 years

**Fig. 4:**
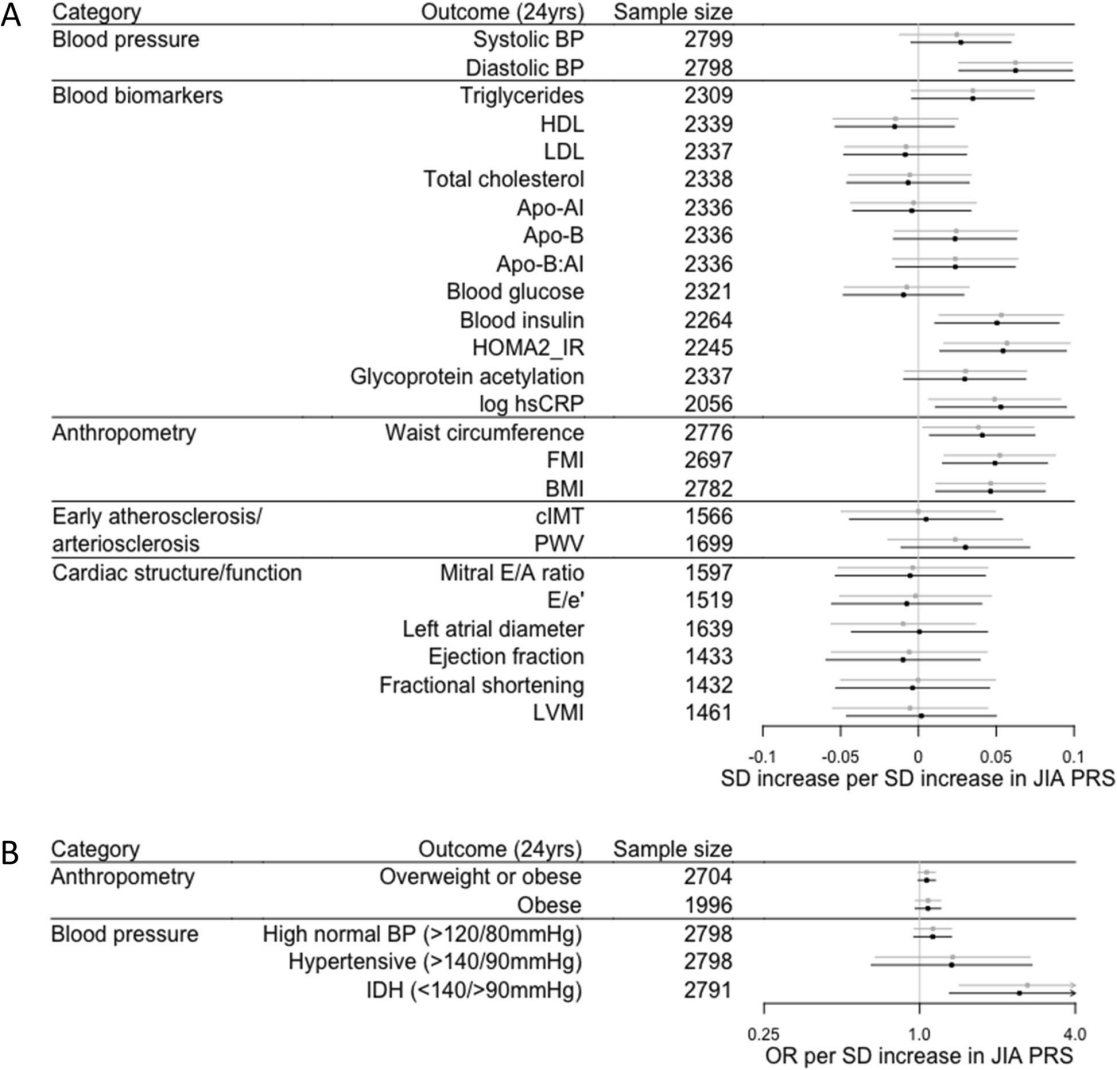
Association between JIA PRS, and **A** continuous and **B** dichotomous cardiovascular phenotypes at age 24 years. Estimates are presented as unadjusted (grey) and adjusted for 10 principal components and sex (black). Apo-AI, apolipoprotein AI; Apo-B, apolipoprotein B; BMI, body mass index; BP, blood pressure; cIMT, carotid intima media thickness; FMI, fat mass index; HDL, high density lipoprotein cholesterol; HOMA2_IR, Homeostasis Model Assessment 2 insulin resistance index; hsCRP, high sensitivity C-reactive protein; IDH, isolated diastolic hypertension; JIA, juvenile idiopathic arthritis; LDL, low density lipoprotein cholesterol; LVMI, left ventricular mass index; PRS, polygenic risk score; SD standard deviation

We observed strong evidence of an association between JIA PRS and multiple, continuous cardiovascular risk factors (Fig. 4, Supplementary Table 5) – increasing JIA PRS was associated with higher diastolic BP (β 0.062, 95% CI 0.026 to 0.099, *P* = 0.001), higher blood insulin levels (β 0.050, 95% CI 0.011 to 0.090, *P* = 0.013), higher HOMA2_IR (β 0.054, 95% CI 0.014 to 0.095, *P* = 0.009), higher log hsCRP (β 0.053, 95% CI 0.011 to 0.095, *P* = 0.014), higher waist circumference (β 0.041, 95% CI 0.007 to 0.075, *P* = 0.017), higher FMI (β 0.049, 95% CI 0.016 to 0.083, *P* = 0.004) and higher BMI (β 0.046, 95% CI 0.011 to 0.081, *P* = 0.010). A suggestive association between other adverse continuous cardiovascular risk factors and JIA PRS was also observed, including higher systolic BP, higher triglycerides, lower HDL, higher Apo-B, higher Apo-B:AI, higher glycoprotein acetylation and faster pulse wave velocity. Similar trends were also seen for dichotomous cardiovascular risk factors; increasing JIA PRS was association with a tendency towards being overweight and/or obese, and abnormal BP measures (Fig. 4, Supplementary Table 6).

#### Sensitivity analyses

To assess the reliability of these findings we undertook several sensitivity analyses. Firstly, to ensure the findings were not being driven by the MHC region, we excluded the single MHC SNP (rs115649989) from the analysis. Effect estimates were largely unchanged (Supplementary Table 7). Secondly, we assessed whether the findings were being driven by the subset of participants who already had a diagnosis of an autoimmune disorder (i.e. those participants contributing to the positive control analysis). Our findings were consistent when those 117 participants were removed from the cohort (Supplementary Table 7). Finally, we also assessed the associations between JIA PRSs derived using different SNP *P* value thresholds (from ≤ 0.01 to ≤ 5 × 10^–8^, Supplementary Figs. 1 and 2) and each outcome. Here, the direction of effect was largely consistent across PRS *P* value thresholds.

### Association between JIA PRS and cardiovascular phenotypes across childhood, adolescence, and early adulthood

For seven outcome variables with strong evidence of association with JIA PRS at age 24 years we performed a longitudinal analysis to assess the association between JIA PRS and each of these outcome variables across childhood from age 7 years to age 24 years. The descriptive statistics for each outcome at each timepoint are in Supplementary Table 8.

**Fig. 5:**
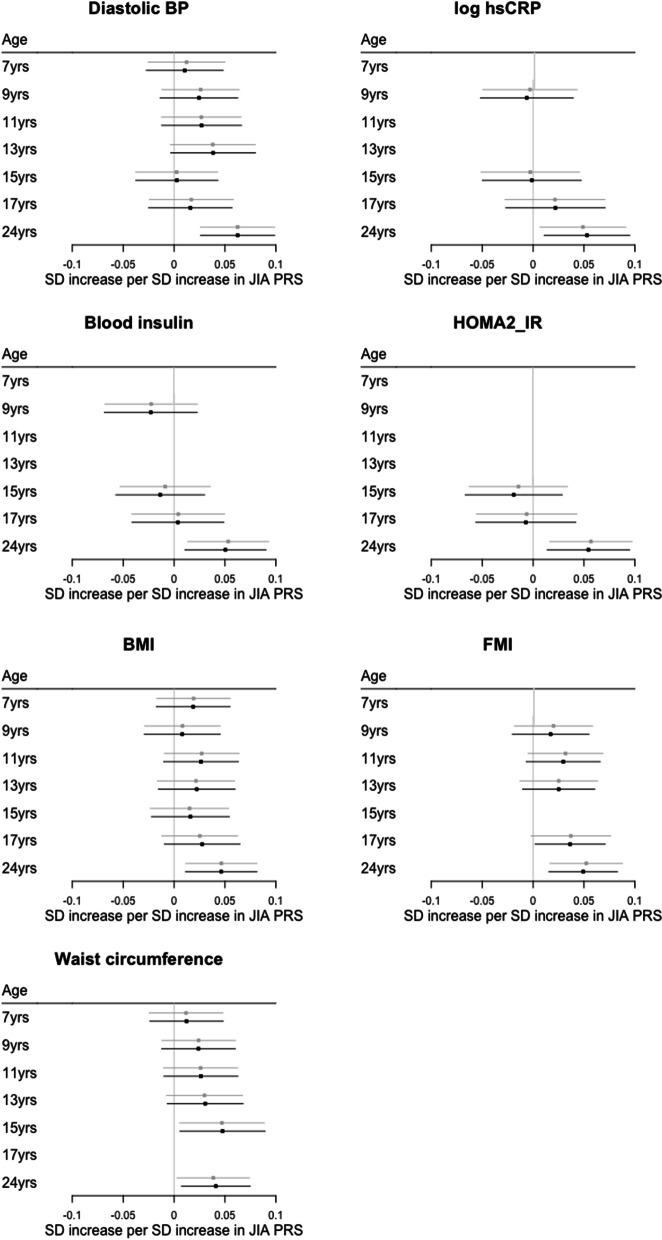
Association between JIA PRS and cardiovascular phenotypes from age 7 years to age 24 years. Estimates are presented as unadjusted (grey) and adjusted for 10 principal components and sex (black). BMI, body mass index; BP, blood pressure; FMI, fat mass index; HOMA2_IR, Homeostasis Model Assessment 2 insulin resistance index; hsCRP, high sensitivity C-reactive protein; JIA, juvenile idiopathic arthritis; SD standard deviation

Using the available measures, we found no strong evidence of an association between JIA PRS and log hsCRP, insulin and HOMA2_IR prior to age 24 years (Fig. 5, Supplementary Table 9). For waist circumference, BMI and FMI, evidence of an association between JIA PRS and an increase in each variable was suggested from age 7 years with the strength and size of these associations increasing with age (Fig. 5, Supplementary Table 9). A similar pattern was seen for diastolic BP with attenuation of the association in the late teenage years (Fig. 5, Supplementary Table 9). These findings were consistent when only the 1,788 participants who attended every clinic visit over the 17-year period were included (Supplementary Table 10).

## Discussion

Using a PRS approach we have shown that genetic liability to JIA is robustly positively associated with multiple cardiovascular risk factors at age 24 years (diastolic BP, blood insulin levels, insulin resistance index, log hsCRP, waist circumference, BMI and FMI), with evidence that some of these associations begin to manifest much earlier in life. We did not find strong evidence of association between genetic liability to JIA and markers of early atherosclerosis/arteriosclerosis or measures of cardiac structure and function at age 24 years, however this may be due to insufficient cumulative exposure to cardiovascular risk factors by early adulthood. The suggestive evidence that increasing genetic liability to JIA is associated with higher PWV at age 24 years supports this view; PWV is correlated with diastolic BP [31] and CRP [32] (both outcomes which are strongly associated with the JIA PRS in this study). Thus, genetic liability to JIA may show associations with other markers of atherosclerosis/arteriosclerosis or cardiac structure and function later in life, as cumulative exposure to cardiovascular risk factors increases. Collectively, our findings support the hypothesis that JIA is associated with adverse cardiovascular risk.

JIA is a highly heritable condition and the clinical application of using a JIA PRS in aiding diagnosis and subtype prediction has recently been reported [33]. However our study is, to our knowledge, the first to examine the utility of JIA PRS in disease prognosis. Our findings of the shared aetiology of JIA and cardiovascular traits at the genetic level has several clinical implications. Firstly, that any cardiovascular impacts of JIA should be regarded as a core feature of the disease rather than a phenomenon secondary to arthritis activity or treatment. Secondly, that cardiovascular risk factor assessment and counselling should be considered in this patient group from an early age. Finally, European guidance exists for the management of cardiovascular risk in adult-onset inflammatory joint diseases (RA, ankylosing spondylitis and psoriatic arthritis) but this guidance does not include paediatric-onset disorders, such as JIA. Consideration should be given as to whether patients with JIA should be included within this guidance or whether bespoke guidance is needed in the context of a paediatric-onset disease.

Further work to examine the trajectories of cardiovascular risk factors in JIA compared to the general population, the association with cardiovascular disease end points (e.g. myocardial infarction), and the impact and timing of primary preventative strategies is also needed. Whilst therapies to alter cardiovascular risk factors e.g. anti-hypertensives and statins are licenced for use in children, the design of interventional studies to examine their efficacy and long-term safety in this context needs careful consideration. In SLE, CVD burden in paediatric-onset disease has been shown to be comparable to those with adult-onset disease [34] however attempts to modify cardiovascular risk in children with SLE have had limited success. The APPLE study [35] examined the role of atorvastatin in preventing subclinical atherosclerosis progression in paediatric-onset SLE, but the study failed to reach its primary end point of reduced cIMT progression. Secondary analysis identified a potential role for atorvastatin in reducing cIMT progression in a subset of patients based on pubertal status and hsCRP [36]. However, the appropriateness of measuring cIMT in young people has been questioned [37] and the influence of blood lipid profiles on cardiovascular risk in the context of inflammatory disorders is not straightforward. Increased total cholesterol, increased LDL levels and decreased HDL levels are associated with cardiovascular risk in healthy individuals [3]. However, in RA, patients with active disease exhibit reduced total cholesterol, HDL and LDL levels [38]. This lipid paradox leads to a complex, U-shaped relationship between cholesterol and cardiovascular risk in RA, which is thought to result from excessive systemic inflammation altering the qualitative function of the lipids in addition to quantitative changes [39]. Furthermore, anti-inflammatory therapies, in addition to reducing systemic inflammation, also increase total cholesterol, HDL and LDL to variable degrees and may alter their function [38]. Lack of strong evidence of an association between genetic liability to JIA and lipids measures in our study may reflect a similarly complex relationship and thus the role of lipid lowering therapies versus anti-inflammatory medications and optimal disease control in JIA requires further research. The timing of such intervention also needs to be considered. In keeping with the secondary analysis from the APPLE study, our study found that the association between JIA PRS and many cardiovascular phenotypes only became evident after adolescence, suggesting that pharmaceutical interventions may be better targeted to young adults rather than during childhood.

We have also shown that phenotypes associated with body habitus (BMI, FMI and waist circumference) associate with JIA PRS at the youngest age. Central adiposity is a well-established independent risk factor for CVD [40] and is itself a low-grade inflammatory state with adipose tissue releasing a number of inflammatory mediators [41]. Conversely, chronic systemic inflammation can reduce lean body mass, predisposing to adiposity [42]. In the context of an inflammatory disorder, careful consideration needs to be given to the influence systemic inflammation has on body habitus, and whether cardiovascular risk associated with JIA is directly mediated by central adiposity, underlying inflammation, other disease-associated factors such as reduced physical activity, or a combination of factors. RA patients with obesity have been shown to be more treatment resistant than those with a normal BMI [43]. In JIA, higher levels of systemic inflammation are associated with increased BMI, FMI and total body fat, with overweight/obese patients having a longer duration of disease, higher hsCRP levels, longer duration of biologic therapy and lower physical activity than those with a normal BMI [44]. Conversely, JIA patients with low disease activity have comparable body composition and anthropometric measures to healthy controls. Decreasing prevalence of overweight/obese JIA patients has also been reported in parallel with good disease control [45]. Physical activity remains a potential mediator of the association between JIA PRS and inflammation and anthropometric measures. JIA patients are reported to have lower levels of physical activity than their heathy peers however the level of physical activity is not consistently associated with physician assessed disease activity [46]. Further work to understand the direct and indirect relationships between JIA PRS, systemic inflammation, anthropometry and physical activity would be helpful in order to counsel patients. Nevertheless, in keeping with the guidance for RA patients [7] optimal disease control and increased physical activity are likely to be important for cardiovascular health in JIA patients, particularly due to the interplay between these factors.

Given the currently limited evidence base regarding the magnitude of CVD risk and optimal strategy for mitigation (particularly with regards to method, timing, benefit and risk of potential pharmaceutical interventions), the clinician’s role in counselling patients about good health behaviours (e.g. smoking cessation, increased physical activity and healthy diet) becomes paramount.

### Strengths and limitations

The major strengths of this study come from the datasets used to examine the association between genetic liability to JIA and cardiovascular phenotypes. The JIA GWAS dataset used in this study is the most recent and most inclusive JIA GWAS available, with a total sample size of 12,501 participants. In keeping with previous studies, this JIA GWAS showed JIA to be a highly heritable condition with SNP-based heritability estimated to be 0.61 [22]. High heritability improves the likely predictive value of the PRS. ALSPAC is a large, well-characterised birth cohort with regular follow up. Data were collected from large numbers of participants using standardised protocols. At the analysis stage, we employed careful study design, including a priori selection of the *P* value threshold for SNP inclusion in the PRS, to minimise bias. The validity of the PRS for JIA was assessed using positive and negative control data based on self-report at the outset, prior to examining the association with cardiovascular phenotypes. The positive association between genetic liability to JIA and markers of systemic inflammation (log hsCRP and glycoprotein acetylation) further supports its validity. All associations were examined using both unadjusted estimates and estimates adjusted for key covariates (genetic ancestry and sex). We also undertook multiple sensitivity analyses to examine the robustness of the main analysis; removing the MHC region, excluding participants with autoimmune disorders, and analyses using PRSs derived using higher and lower stringency *P* value thresholds.

Nevertheless, our study also has some limitations. It is likely that the degree of systemic inflammation varies by JIA subtype (for example systemic versus oligoarticular JIA) and that, as a result, the magnitude of association with cardiovascular phenotypes may also vary by subtype. The current lack of subtype specific JIA GWAS data prevents assessment of subtype specific associations with cardiovascular phenotypes. One source of bias within the study is the representativeness of the ALSPAC cohort. As is the case with most longitudinal cohorts, there has been attrition of participants in the ALSPAC study over time. This has led to under-representation of participants from lower socio-economic groups [47]. Additionally, participation in ALSPAC has also been shown to be influenced by genetic liability to multiple lifestyle factors, personal characteristics and health outcomes [21]. Nevertheless children in the ALSPAC cohort have been shown to be representative of the general population in terms of birth weight and birth length [48]. We were unable to directly assess the ability of the JIA PRS to discriminate JIA case versus control status as there is no specific measure of JIA status in ALSPAC. To address this, we used a positive and negative control design to evaluate the performance of the JIA PRS prior to examining the association with cardiovascular phenotypes. As a positive control variable, we exploited the genetic overlap between autoimmune disorders and examined the association between JIA PRS and diagnosis of autoimmunity by age 24 years. We did not identify strong evidence of a positive association; however, the positive control variable captures a highly heterogenous group of autoimmune disorders. Furthermore, the positive control variable is subject to misclassification bias in the control group; ALSPAC participants may still develop autoimmune disorders after the age of 24 years. Given these limitations, suggestive evidence of a positive association between JIA PRS and diagnosis of an autoimmune disorder by age 24 years at a *P* value threshold of 1 × 10^–5^ was deemed sufficient. Finally, this study examines genetic liability to JIA rather than JIA itself and thus we are unable to examine the influence of other JIA-related variables such as disease duration, disease activity and treatment modality on cardiovascular phenotypes.

## Conclusion

To our knowledge this is the first study to examine the association between genetic liability to JIA and multiple cardiovascular phenotypes, across childhood and into early adulthood. This study suggests that cardiovascular risk in JIA is not solely secondary to disease duration, disease activity or effects of therapy, and that cardiovascular risk should be considered a core component of the disease. This study has implications for clinical care and underscores the need for cardiovascular risk assessment and counselling in patients with JIA. Further work is required to examine the optimal timing for assessment of cardiovascular risk in JIA and the utility of cardiovascular risk factor modification in this population.

## Supplementary Information

**Additional file 1: Supplementary methods.**

**Additional file 2: Supplementary Table 1.** Number of SNPs included in each JIA PRS generated using different SNP *P* value thresholds. **Supplementary Table 2.** SNPs included in the JIA PRS with the SNP *P* value threshold of <1.00E-5. SE, standard error; SNP, single nucleotide polymorphism. **Supplementary Table 3.** Data inclusion and data losses for the ALSPAC cohort and associated cardiovascular phenotypes. Outlying data was defined as those greater than three times the interquartile range. To maintain data anonymity, any cell containing a value less than 5 (including 0) has been replaced with <5. Apo-AI, apolioprotein-AI; Apo-B, apolipoprotein-B; BMI, body mass index; BP, Blood pressure; cIMT, carotid intima-media thickness; hsCRP, high sensitivity C-reactive protein; FMI, fat mass index; HOMA2_IR, Homeostasis Model Assessment 2 insulin reistance index; HDL, high density lipoprotein cholesterol; LDL, low density lipoprotein cholesterol; LVMI, left ventricular mass index. **Supplementary Table 4.** Assocations between JIA PRS generated using SNPs with the *P* value <5e-5, and positive and negative control outcomes. Pseudo R2 is used to evaulate the gooness of fit. CI, confidence interval; JIA, JIA; OR, odds ratio; PRS, polygenic risk score. **Supplementary Table 5.** Assocations between JIA PRS generated using SNPs with the *P* value <5e-5 and continous cardiovascular outcomes. *adjusted for 10 ancestry informed principal components and sex. Outcomes with *P*<0.05 are italicised. R2 is the proportion of the variance in the outcome which can be explained by the JIA PRS. Apo-AI, apolioprotein-AI; Apo-B, apolipoprotein-B; BMI, body mass index; BP, Blood pressure; CI, confidence interval; cIMT, carotid intima-media thickness; hsCRP, high sensitivity C-reactive protein; FMI, fat mass index; HOMA2_IR, Homeostasis Model Assessment 2 insulin reistance index; HDL, high density lipoprotein cholesterol; JIA, juvenile idiopathic arthritis; LDL, low density lipoprotein cholesterol; LVMI, left ventricular mass index; PRS, polygenic risk score. **Supplementary Table 6.** Assocations between JIA PRS generated using SNPs with the *P* value <5e-5 and dichotomous cardiovascular outcomes. *adjusted for 10 ancestry informed principal components and sex. Outcomes with *P*<0.05 are italicised. Pseudo R2 is used to evaulate the gooness of fit. BP, blood pressure; CI, confidence interval; JIA, juvenile idiopathic arthritis; OR, odds ratio. **Supplementary Table 7.** Assocations between JIA PRS generated using SNPs with the *P* value <1e-5 and cardiovascular outcomes in the main analysis, sensitivity analysis 1 (exclusion of MHSCregion SNP) and sensitivity analysis 2 (exclusion of participants with autoimmune disease). All estimates are adjusted for 10 ancestry informed principal components and sex. Outcomes with *P* <0.05 are italicised. *For dichotomous outcomes, OR (95% CI) are reported. R2 is the proportion of the variance in the outcome which can be explained by the JIA PRS. ^for dichotomous outcomes, pseudo R2 is reported. Pseudo R2 is used to evaulate the gooness of fit. Apo-AI, apolioprotein-AI; Apo-B, apolipoprotein-B; BMI, body mass index; BP, Blood pressure; CI, confidence interval; cIMT, carotid intima-media thickness; hsCRP, high sensitivity C-reactive protein; FMI, fat mass index; HOMA2_IR, Homeostasis Model Assessment 2 insulin reistance index; HDL, high density lipoprotein cholesterol; JIA, juvenile idiopathic arthritis; LDL, low density lipoprotein cholesterol; LVMI, left ventricular mass index; PRS, polygenic risk score. **Supplementary Table 8.** Assocations between JIA PRS generated using SNPs with the *P* value <5e-5 and cardiovascular outcomes from age 7 years to age 24 years for participants who attended the Focus24+ clinic. ^For measures which are not continuous N (%) is reported and participants with missing data are excluded from the denominator. *variable not calculated for earlier time points due to the different clinical thresholds by age. Apo-AI, apolioprotein-AI; Apo-B, apolipoprotein-B; BMI, body mass index; BP, Blood pressure; cIMT, carotid intima-media thickness; hsCRP, high sensitivity C-reactive protein; FMI, fat mass index; HOMA2_IR, Homeostasis Model Assessment 2 insulin reistance index; HDL, high density lipoprotein cholesterol; LDL, low density lipoprotein cholesterol; LVMI, left ventricular mass index. **Supplementary Table 9.** Assocations between JIA PRS generated using SNPs with the *P* value <5e-5 and cardiovascular outcomes from age 7 years to age 24 years. *adjusted for 10 ancestry informed principal components and sex. Outcomes with *P*<0.05 are italicised. R2 is the proportion of the variance in the outcome which can be explained by the JIA PRS. BMI, body mass index; BP, Blood pressure; CI, confidence interval; hsCRP, high sensitivity C-reactive protein; FMI, fat mass index; HOMA2_IR, Homeostasis Model Assessment 2 insulin reistance index; IA, juvenile idiopathic arthritis; PRS, polygenic risk score. **Supplementary Table 10.** Assocations between JIA PRS generated using SNPs with the *P* value <5e-5 and cardiovascular outcomes from age 7 years to age 24 years for Focus24+ participants who attended a) any earlier clinic and b) every earlier clinic. All estiamtes are adjusted for 10 ancestry informed principal components and sex. R2 is the proportion of the variance in the outcome which can be explained by the JIA PRS. Sample size varies by outcome due to missing data. BMI, body mass index; BP, Blood pressure; CI, confidence interval; hsCRP, high sensitivity C-reactive protein; FMI, fat mass index; HOMA2_IR, Homeostasis Model Assessment 2 insulin reistance index; IA, juvenile idiopathic arthritis; PRS, polygenic risk score.

**Additional file 3:** **Supplementary figure 1.** Association between JIA PRS and continuous cardiovascular outcomes at age 24 years at *P* value thresholds between <0.01 to <5x10-8. Apo-AI, apolipoprotein AI; Apo-B, apolipoprotein B; BMI, body mass index; BP, blood pressure; cIMT, carotid intima media thickness; FMI, fat mass index; HDL, high density lipoprotein cholesterol; HOMA2_IR, Homeostasis Model Assessment 2 insulin resistance index; hsCRP, high sensitivity C-reactive protein; IDH, isolated diastolic hypertension; JIA, juvenile idiopathic arthritis; LDL, low density lipoprotein cholesterol; LVMI, left ventricular mass index; PRS, polygenic risk score; SD standard deviation. **Supplementary figure 2.** Association between JIA PRS and dichotomous cardiovascular outcomes at age 24 years at *P* value thresholds between <0.01 to <5x10-8. IDH, isolated diastolic hypertension; JIA, juvenile idiopathic arthritis; OR, odds ratio; PRS, polygenic risk score; SD standard deviation.

## Data Availability

The informed consent obtained from ALSPAC participants does not allow the data to be made freely available through any third party maintained public repository. However, data used for this submission can be made available on request to the ALSPAC Executive. The ALSPAC data management plan describes in detail the policy regarding data sharing, which is through a system of managed open access. Full instructions for applying for data access can be found here: http://www.bristol.ac.uk/alspac/researchers/access/. The ALSPAC study website contains details of all the data that are available (http://www.bristol.ac.uk/alspac/researchers/our-data/). A version of the R script used in this analysis is available at https://github.com/sc3170/JIA-polygenic-risk-and-cardiovascular-phenotypes.

## Abbreviations

ALSPAC: Avon Longitudinal Study of Parents and Children
Apo-AI: Apolipoprotein AI
Apo-B: Apolipoprotein B
BP: Blood pressure
BMI: Body mass index
CI: Confidence interval
cIMT: Carotid intima media thickness
CVD: Cardiovascular disease
EF: Ejection fraction
FMI: Fat mass index
FS: Fractional shortening
GWAS: Genome wide association study
HDL: High density lipoprotein cholesterol
HOMA2_IR: Homeostasis Model Assessment 2 insulin resistance index
hsCRP: High sensitivity C-reactive protein
JIA: Juvenile idiopathic arthritis
LD: Linkage disequilibrium
LDL: Low density lipoprotein cholesterol
LVM: Left ventricular mass index
MHC: Major histocompatibility complex
PRS: Polygenic risk score
PWV: Pulse wave velocity
RA: Rheumatoid arthritis
SD: Standard deviation
SLE: Systemic lupus erythematosus
