# Supplementary material for: Juvenile idiopathic arthritis polygenic risk scores are associated with cardiovascular phenotypes in early adulthood: a phenome-wide association study
Source: Pediatr Rheumatol Online J. 2022 Nov 19;20:105. doi: 10.1186/s12969-022-00760-0 (PMC9675123; doi:10.1186/s12969-022-00760-0)
Supplement: Supplementary file 1 — Additional file 1: Supplementary methods. [file 12969_2022_760_MOESM1_ESM.docx]

**Supplementary methods**

**JIA genetic data**

The JIA genetic data used to generate the weights for the polygenic risk scores (PRSs) in this study derives from a recent study by Lopez-Isac et al.^1^ Briefly, JIA samples were genotyped on the Illumina Infinium CoreExome and Infinium OmniExpress genotyping arrays and healthy controls were genotyped on the Illumina Infinium CoreExome genotyping array. Quality controls processes excluded SNPs with call rate <0.98, minor allele frequency <0.01 and non-autosomal SNPs. Samples with discrepancy between recorded and genetically inferred sex were also excluded. Related individuals were identified using identity-by-descent, and the sample with the highest call rate was retained. Ancestral outliers were identified and excluded based on principal component analysis. Phasing and imputation were carried out using SHAPEIT2 and Minimac3, using the Haplotype Reference Consortium reference panel. Following imputation, SNPs were excluded based on MAF <0.01 and imputation quality (r^2^) <0.4.

Following QC, the total dataset comprised 3,305 JIA patients and 9,196 healthy controls.

**ALSPAC genetic dataset**

ALSPAC children were genotyped using the Illumina HumanHap550 quad genome-wide single nucleotide polymorphism (SNP) genotyping platform (23andme subcontracting the Wellcome Trust Sanger Institute, Cambridge, UK and the Laboratory Corporation of America, Burlington, NC, US). The raw data was then quality controlled according to the following process using PLINK (v1.07).^2,3^ Individuals were excluded due to gender mismatch, minimal or excessive heterozygosity, level of individual missingness (>3%), and insufficient sample replication (IBD <0.8). Population stratification was assessed using multidimensional scaling analysis and compared with Hapmap II (release 22) European descent (CEU), Han Chinese, Japanese and Yoruba reference populations. All individuals with non-European ancestry were removed. SNPs were removed which had minor allele frequency <1%, a call rate of <95% or evidence of violations of the Hardy-Weinberg equilibrium (P <5x10^-7^). Cryptic relatedness was measured as proportion of identity by descent (IBD > 0.1). Related subjects that passed all other quality control thresholds were retained during subsequent phasing and imputation. 9,115 subjects and 500,527 SNPs passed these quality control filters.^4^

Following QC and imputation, the total dataset comprised 7,977 ALSPAC participants.

**ALSPAC outcome variables**

**Anthropometric measures**

Participant weight was measured every two years from age seven years to age 17 years and again at age 24 years using a Tanita Body Fat Analyser weighing scale (Tanita, West Drayton, Middlesex, UK). Weight measured were recorded to the nearest 0.1kg. Standing height, without shoes, was measured from age seven years to age 17 years and again at age 24 years using a Harpenden wall-mounted stadiometer (Holtain Ltd, Crymych, Pembs, UK). Height was recorded to the nearest 1mm. Waist circumference at the midpoint between the lower ribs and the iliac crest was measured every 2 years from age seven years to age 15 years and again at age 24 years. Measurements were taken using a flexible tape and recorded to the nearest 1mm. Average waist circumference was calculated as the mean of two repeated waist circumference measures; where only one measurement was taken, that value was used. BMI was calculated every two years from age seven years to age 17 years and again at age 24 years, using the formula $BMI=\frac{weight \left( kg \right)}{height ({m)}^{2}}$. For the age 17 years and age 24 years clinics, BMI estimates were categorised as underweight (BMI <18.5kg/m^2^), normal (BMI 18.5-24.9 kg/m^2^), overweight (BMI 25.0-29.9 kg/m^2^), obese (BMI >30 kg/m^2^). For earlier time points (ages 7 to 15 years) standard deviation (SD) scores were calculated using the *sdschild* R package^5^ and the UK-WHO reference ranges.^6^ Standard deviation scores were then used to categorise BMI according to the WHO BMI-for-age thresholds^7,8^ as underweight (< -2 SD), normal (> -2 SD to < +1 SD), overweight (>1 SD to < +2 SD) and obese (> +2 SD). BMI was assessed as both a continuous and a categorical variable within this study. Total body fat mass was assessed every two years from age nine years to age 15 years and again at age 17 years and 24 years using Lunar Prodigy DEXA scanner (GE Medical Systems Lunar, Madison, WI, USA). Participants were excluded from scanning if they a) were pregnant at the time of scan, b) had had a radiological investigation using contrast media in the previous week, c) had had recent nuclear medicine investigation with persistent radioactivity or d) weighed more than 159kg. FMI was calculated as $FMI=\frac{FM (kg)}{height {(m)}^{2}}$.

**Blood pressure**

Blood pressures measures were taken every two years from age seven years to age 17 years and again at age 24 years. A Dinamap 9301 Vital Signs Monitor was used for the age 7, 9, 11 and 17 year clinic visits, a Dinamap 8100 Vital Signs Monitor for the age 13 years clinic visit, and an Omron IntelliSense M6 monitor for the age 15 years and 24 years clinic visits.. All measures were taken after two minutes of rest, with the participant in a seated position, using an appropriately sized cuff, and with the arm supported. Measurements were repeated up to three times depending on the timing of clinic visit. Average seated systolic and diastolic blood pressure values were calculated from the available systolic and diastolic blood pressure measures at each time point.

**Blood measures**

*Clinical biochemistry*

Blood samples taken at age nine years were non-fasting. For samples taken at later time points, participants fasted overnight or for at least six hours prior to their clinic visit. Samples were centrifuged immediately after collection and stored at -80°C. Samples were analysed three to nine months later with no record of previous freeze–thaw cycles. Total cholesterol (TC, mmol/L), low density lipoprotein cholesterol (LDL, mmol/L), high density lipoprotein cholesterol (HDL, mmol/L) and triglycerides (TG, mmol/L) were measured using the standard Lipid Research Clinics Protocol using enzymatic reagents for lipid determination. LDL concentration was calculated using the Friedewald equation.^9^ Insulin (μIU/mL) was measured using an ELISA immunoassay with no cross-reactivity with pro-insulin up to 1000 pmol/l (Mercodia). High sensitivity CRP (mg/L) was measured using an automated particle-enhanced immunoturbidimetric assay (Roche UK, Welwyn Garden City, UK). Homeostasis model assessment score for insulin resistance index was calculated using the HOMA2 calculator v2.2.4 (HOMA2-IR, Diabetes Trials Unit, Oxford). Measures of fasting insulin were converted from μIU/mL to pmol/L using a conversion factor of 1 μIU/mL is equal to 6.00 pmol/L.

*Metabolomic assays*

At age 9, 15 and 17 metabolomic assays were performed using high-throughput proton (^1^H) Nuclear magnetic resonance (NMR) spectroscopy-based platform (Nightingale Health, Helsinki, Finland) on plasma samples as has previously described.^10-13^ Samples taken at age 9 years were non-fasting. For samples taken at later time points, participants fasted overnight or for at least six hours prior to their clinic visit. The blood metabolites assessed using these NMR platforms were glycoprotein acetylation (mainly a1-acid glycoprotein, mmol/L), glucose (mmol/L), apolipoprotein A-I (g/L), apolipoprotein B (g/L), ratio of apolipoprotein B to A-I.

**Markers of early atherosclerosis/arteriosclerosis**

*Carotid intima media thickness (cIMT)*

At age 24yrs left and right cIMT was measured in end-diastole at the far wall of the distal 1 cm of the common carotid arteries (immediately proximal to the bulb) using B-mode ultrasound with the participant supine, neck partially extended and the head rotated to 45 degrees from the midline.

Automated measures of the left and right cIMT were obtained over three cardiac cycles using a CardioHealth ultrasound machine (Panasonic) equipped with a 13.5MHz linear array broadband transducer. All measurements >1.0mm were reviewed by trained personnel. Average cIMT was derived by averaging the left and right cIMT measures; here only a left or right cIMT measure was available, that measure was used.

*Pulse wave velocity (PWV)*

PWV was measured between the carotid and femoral arteries at 24yrs using a Vicorder instrument (Skidmore Medical). Pressure sensor cuffs were placed on the neck (sensor over the right carotid artery) and top of right leg (sensor over femoral artery). The distance between the suprasternal notch directly to the top of the thigh cuff and the suprasternal notch to the bottom of the neck cuff were measured to the nearest millimetre. PWV measurements were repeated until three measurements were obtained that were within 0.5m/sec of one another, or until a total of 5 measurements had been made.

**Echocardiographic measures**

*Baseline measures*

Echocardiography were performed on study participants at age 24 years with a Philips EPIQ 7G Ultrasound System equipped with a X5-1 xMATRIX array transducer. M-mode, 2D and Doppler echo analyses were performed using Philips Q-station. Three dimensional echocardiogram analyses were performed using TomTec software. All measurements were obtained based on American Society of Echocardiography guidance.^14,15^

The following measures were extracted from the ALSPAC dataset and used to derive measures of cardiac structure and function at age 24 years: mitral A wave peak (A), mitral E wave peak (E), left ventricle lateral wall e’ velocity (e’_lateral_), left ventricle septal wall e’ velocity (e’_septal_), interventricular septum thickness in diastole (IVS_d_), posterior wall thickness in diastole (PW_d_), left ventricle internal diameter in diastole (LVID_d_) and left ventricle internal diameter in systolic (LVID_s_).

*Systolic function*

Systolic function was assessed by ejection fraction (EF) and fractional shortening (FS). EF was determined by $EF \left( \% \right)=(V_{d}-V_{s})/V_{d}\times100$ where $V_{d}=(\frac{7}{2.4+{LVID}_{d}})\times{LVID}_{d}^{3}$ and $V_{s}=(\frac{7}{2.4+{LVID}_{s}})\times{LVID}_{s}^{3}$. FS (%) was determined by $FS \left( \% \right)=({LVID}_{d}-{LVID}_{s})/{LVID}_{d}\times100$.

*Diastolic function*

Diastolic function was assessed using E/A ratio, E/e’ ratio and left atrial diameter. E/e’ is calculated as $E/e'=E/(\frac{{e^{'}}_{lateral}+{e^{'}}_{septal}}{2})$.

*Left ventricular mass (LVM) index*

LVM is calculated using the Deveureux’s formula^16^ $LVM (g)=0.8\times(1.04\times(\left( {IVS}_{d}+{LVID}_{d}+{{PW}_{d})}^{3}-{LVID}_{d}^{3} \right))+0.6$. LVM was normalised to height^2.7^, as this method has been shown to correlated well with lean body mass and excludes the effect of obesity and BP elevation on left ventricular mass in older children and adolescents.^17-19^

For all cardiovascular imaging studies (echocardiography, cIMT and PWV) data were checked for outlying or abnormal values by a trained research scientist working on the project. Abnormal values due to measurement error were removed. Pregnant participants were also excluded from the cardiovascular imaging studies

References

1. E Lopez-Isac, SL Smith, MC Marion, A Wood, M Sudman, A Yarwood, et al. Combined genetic analysis of juvenile idiopathic arthritis clinical subtypes identifies novel risk loci, target genes and key regulatory mechanisms. Ann Rheum Dis. 2020. doi: 10.1136/annrheumdis-2020-218481

2. S Purcell. PLINK (v1.07). Web site: <http://pngu.mgh.harvard.edu/purcell/plink/>. Accessed October 28,, 2021.

3. S Purcell, B Neale, K Todd-Brown, L Thomas, MA Ferreira, D Bender, et al. PLINK: a tool set for whole-genome association and population-based linkage analyses. Am J Hum Genet. 2007;81(3):559-75. doi: 10.1086/519795

4. ALSPAC. ALSPAC OMICs Data Catalogue. Web site: <https://alspac.github.io/omics_documentation/alspac_omics_data_catalogue.html#org3f1315c>. Published July 8, 2017. Accessed Oct 26, 2021.

5. Author. Childsds: Data and Methods Around Reference Values in Pediatrics. R package. Journal. 2020 Available from: <https://cran.r-project.org/package=childsds>.

6. CM Wright, AF Williams, D Elliman, H Bedford, E Birks, G Butler, et al. Using the new UK-WHO growth charts. BMJ. 2010;340:c1140. doi: 10.1136/bmj.c1140

7. M de Onis, AW Onyango, E Borghi, A Siyam, C Nishida and J Siekmann. Development of a WHO growth reference for school-aged children and adolescents. Bull World Health Organ. 2007;85(9):660-7. doi: 10.2471/blt.07.043497

8. World Health Organization. BMI-for-age (5-19 years). Web site: <https://www.who.int/tools/growth-reference-data-for-5to19-years/indicators/bmi-for-age>. Accessed 21st october 2021, 2021.

9. WT Friedewald, RI Levy and DS Fredrickson. Estimation of the concentration of low-density lipoprotein cholesterol in plasma, without use of the preparative ultracentrifuge. Clin Chem. 1972;18(6):499-502. doi:

10. M Inouye, J Kettunen, P Soininen, K Silander, S Ripatti, LS Kumpula, et al. Metabonomic, transcriptomic, and genomic variation of a population cohort. Mol Syst Biol. 2010;6:441. doi: 10.1038/msb.2010.93

11. P Soininen, AJ Kangas, P Wurtz, T Suna and M Ala-Korpela. Quantitative serum nuclear magnetic resonance metabolomics in cardiovascular epidemiology and genetics. Circ Cardiovasc Genet. 2015;8(1):192-206. doi: 10.1161/CIRCGENETICS.114.000216

12. P Soininen, AJ Kangas, P Wurtz, T Tukiainen, T Tynkkynen, R Laatikainen, et al. High-throughput serum NMR metabonomics for cost-effective holistic studies on systemic metabolism. Analyst. 2009;134(9):1781-5. doi: 10.1039/b910205a

13. J Kettunen, T Tukiainen, AP Sarin, A Ortega-Alonso, E Tikkanen, LP Lyytikainen, et al. Genome-wide association study identifies multiple loci influencing human serum metabolite levels. Nat Genet. 2012;44(3):269-76. doi: 10.1038/ng.1073

14. RM Lang, LP Badano, V Mor-Avi, J Afilalo, A Armstrong, L Ernande, et al. Recommendations for cardiac chamber quantification by echocardiography in adults: an update from the American Society of Echocardiography and the European Association of Cardiovascular Imaging. J Am Soc Echocardiogr. 2015;28(1):1-39 e14. doi: 10.1016/j.echo.2014.10.003

15. SF Nagueh, OA Smiseth, CP Appleton, BF Byrd, 3rd, H Dokainish, T Edvardsen, et al. Recommendations for the Evaluation of Left Ventricular Diastolic Function by Echocardiography: An Update from the American Society of Echocardiography and the European Association of Cardiovascular Imaging. J Am Soc Echocardiogr. 2016;29(4):277-314. doi: 10.1016/j.echo.2016.01.011

16. RB Devereux. Detection of left ventricular hypertrophy by M-mode echocardiography. Anatomic validation, standardization, and comparison to other methods. Hypertension. 1987;9(2 Pt 2):II19-26. doi: 10.1161/01.hyp.9.2_pt_2.ii19

17. SR Daniels, TR Kimball, JA Morrison, P Khoury and RA Meyer. Indexing left ventricular mass to account for differences in body size in children and adolescents without cardiovascular disease. Am J Cardiol. 1995;76(10):699-701. doi: 10.1016/s0002-9149(99)80200-8

18. G de Simone, SR Daniels, RB Devereux, RA Meyer, MJ Roman, O de Divitiis, et al. Left ventricular mass and body size in normotensive children and adults: assessment of allometric relations and impact of overweight. J Am Coll Cardiol. 1992;20(5):1251-60. doi: 10.1016/0735-1097(92)90385-z

19. SR Daniels, RA Meyer, YC Liang and KE Bove. Echocardiographically determined left ventricular mass index in normal children, adolescents and young adults. J Am Coll Cardiol. 1988;12(3):703-8. doi: 10.1016/s0735-1097(88)80060-3
