# Supplementary material for: Juvenile idiopathic arthritis polygenic risk scores are associated with cardiovascular phenotypes in early adulthood: a phenome-wide association study
Source: Pediatr Rheumatol Online J. 2022 Nov 19;20:105. doi: 10.1186/s12969-022-00760-0 (PMC9675123; doi:10.1186/s12969-022-00760-0)
Supplement: Supplementary file 3 — Additional file 3: Supplementary figure 1. Association between JIA PRS and continuous cardiovascular outcomes at age 24 years at P value thresholds between <0.01 to <5x10^-8^. Apo-AI, apolipoprotein AI; Apo-B, apolipoprotein B; BMI, body mass index; BP, blood pressure; cIMT, carotid intima media thickness; FMI, fat mass index; HDL, high density lipoprotein cholesterol; HOMA2_IR, Homeostasis Model Assessment 2 insulin resistance index; hsCRP, high sensitivity C-reactive protein; IDH, isolated diastolic hypertension; JIA, juvenile idiopathic arthritis; LDL, low density lipoprotein cholesterol; LVMI, left ventricular mass index; PRS, polygenic risk score; SD standard deviation. Supplementary figure 2. Association between JIA PRS and dichotomous cardiovascular outcomes at age 24 years at P value thresholds between <0.01 to <5x10^-8^. IDH, isolated diastolic hypertension; JIA, juvenile idiopathic arthritis; OR, odds ratio; PRS, polygenic risk score; SD standard deviation. [file 12969_2022_760_MOESM3_ESM.docx]

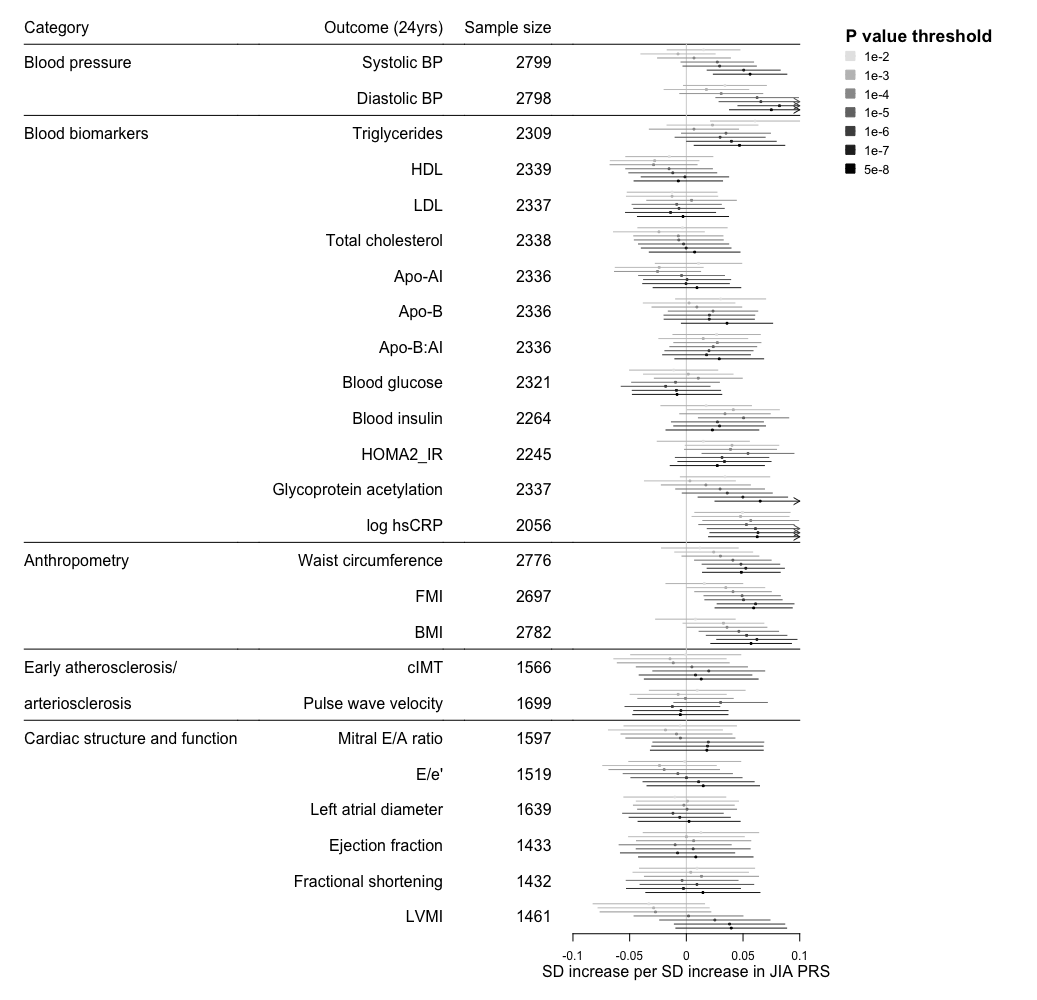


Supplementary figure 1: Association between JIA PRS and continuous cardiovascular outcomes at age 24 years at P value thresholds between <0.01 to <5x10^-8^. Apo-AI, apolipoprotein AI; Apo-B, apolipoprotein B; BMI, body mass index; BP, blood pressure; cIMT, carotid intima media thickness; FMI, fat mass index; HDL, high density lipoprotein cholesterol; HOMA2_IR, Homeostasis Model Assessment 2 insulin resistance index; hsCRP, high sensitivity C-reactive protein; IDH, isolated diastolic hypertension; JIA, juvenile idiopathic arthritis; LDL, low density lipoprotein cholesterol; LVMI, left ventricular mass index; PRS, polygenic risk score; SD standard deviation.


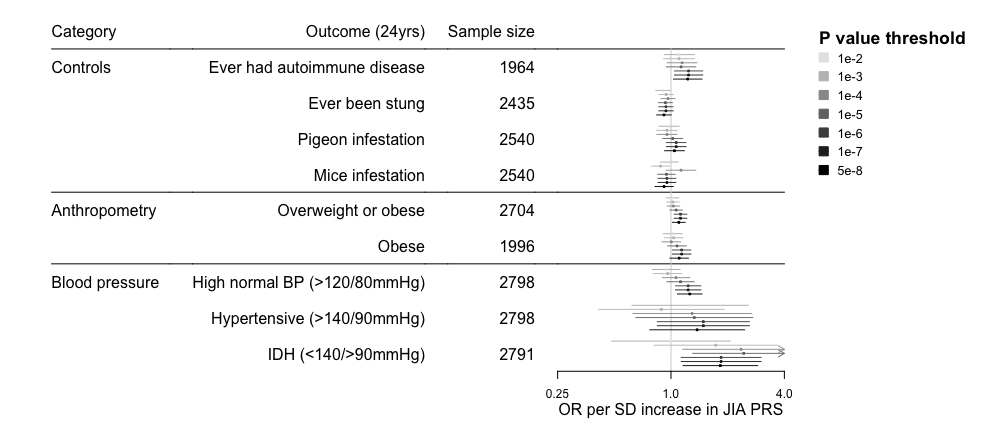


Supplementary figure 2: Association between JIA PRS and dichotomous cardiovascular outcomes at age 24 years at P value thresholds between <0.01 to <5x10^-8^. IDH, isolated diastolic hypertension; JIA, juvenile idiopathic arthritis; OR, odds ratio; PRS, polygenic risk score; SD standard deviation.
